# Overabundance of *Veillonella* parvula promotes intestinal inflammation by activating macrophages via LPS-TLR4 pathway

**DOI:** 10.1038/s41420-022-01015-3

**Published:** 2022-05-06

**Authors:** Zhiyan Zhan, Wenxue Liu, Liya Pan, Yiwen Bao, Zhilong Yan, Li Hong

**Affiliations:** 1 Department of Clinical Nutrition, Shanghai Children’s Medical Center, School of Medicine, Shanghai Jiao Tong University, Shanghai, 200127 China; 2 Department of Obstetrics and Gynecology, Zhongshan Hospital, Fudan University, Shanghai, China; 3 Department of Surgery, Shanghai Children’s Medical Center, School of Medicine, Shanghai Jiao Tong University, Shanghai, 200127 China

**Keywords:** Chemokines, Dysbiosis

## Abstract

Hirschsprung’s disease-associated enterocolitis (HAEC) is the most common complication of Hirschsprung’s disease (HSCR). The microbiome pattern of intestinal flora in HAEC patients was significantly abnormal compared to that in HSCR patients. The overabundance of *V. parvula* was detected in the gut of HAEC patients. To elucidate the pathological mechanisms of the overabundance of *V. parvula*, we established and analyzed inflammatory models induced by LPS or single-bacterial strain transplantation in vivo. The transplantation of *V. parvula* induced inflammatory response in the colon of mice. Besides, we found that LPS from *V. parvula* can significantly impair the barrier function of colonic epithelial cells and then activate macrophages which impaired pacemaker function of interstitial cells of Cajal (ICCs). It was thus a vicious cycle, where the macrophage-related inflammation caused by *V. parvula* via LPS-TLR4 pathway damaged the intestinal motility, which further aggravated the intestinal flora dysbiosis and promoted the development of HAEC. Itaconic acid could break the vicious cycle by inhibiting the activation of macrophages. It could be a potential therapeutic strategy for HAEC patients with intestinal flora dysbiosis.

## Introduction

Hirschsprung’s disease (HSCR) is a congenital and developmental disease characterized by the absence of ganglion cells in the distal bowel, which results in proximal extension and functional obstruction following severe chronic constipation and abdominal distension [1, 2]. Hirschsprung’s disease-associated enterocolitis (HAEC), the most common and severe complication of HSCR, may lead to death in children without timely and effective treatment [3]. Up to 40% of HSCR patients would still have HAEC after radical operation [4]. So far, a number of genetic and immunological studies on HSCR and HAEC have proposed many etiological hypothesizes, but the pathogenesis of HAEC is still unclear.

The composition and diversity of intestinal microbiome are associated with many diseases including HSCR and HAEC [5–9]. The host and the intestinal flora were found to be interactive [10]. In our previous studies on HAEC, we found that the microbiota in HAEC patients was significantly different from that in HSCR patients, and the specimens of different intestinal sites of each HAEC patient were more similar than those of HSCR patients [5, 6]. We found more *Veillonella* (5%) in the intestinal tract of HAEC patients. *Veillonella* are an anaerobic gram-negative cocci as part of the normal flora in healthy people [11]. *Veillonella* are also opportunistic pathogenic bacteria. The overgrowth of *Veillonella* is associated with many diseases, including oral diseases, endocarditis, primary sclerosing cholangitis and ulcerative colitis [9, 11, 12]. In our previous study, *Veillonella parvula* (*V. parvula*) belonging to *Veillonella* genus was detected to be overabundant in the gut of HAEC patients [6]. However, the pathological mechanisms of the effects of *V. parvula* on colitis are still poorly understood.

It is well known that intestinal tract is not only a digestive organ but also the biggest immune organ with the largest number of immune cells. Recently, a study has found that TNF-α secreted by colonic M1 macrophages could impair the pacemaker function of interstitial cells of Cajal (ICCs) through NF-κB/miR-221 pathway in HAEC, resulting in intestinal dysmotility [13]. Intestinal dysmotility will then lead to fecal accumulation and intestinal flora alteration in HAEC patients. Besides, itaconic acid has recently emerged as an inhibitor of inflammatory response in macrophages by stabling Nrf2 protein. The application of itaconic acid may be a potential therapeutic strategy for HAEC patients with dysbacteriosis.

To investigate the pathological mechanisms of colitis induced by *V. parvula*, we examined the phenotypes of colonic epithelial cells and macrophages in our models in vivo and in vitro. We have also identified the anti-inflammatory effects of itaconic acid on the development of colitis induced by LPS from *V. parvula*, which can be a good choice to improve the condition of HAEC patients.

## Results

### The pro-inflammatory effects of *V. parvula* overabundance in the intestine

**Fig. 1:**
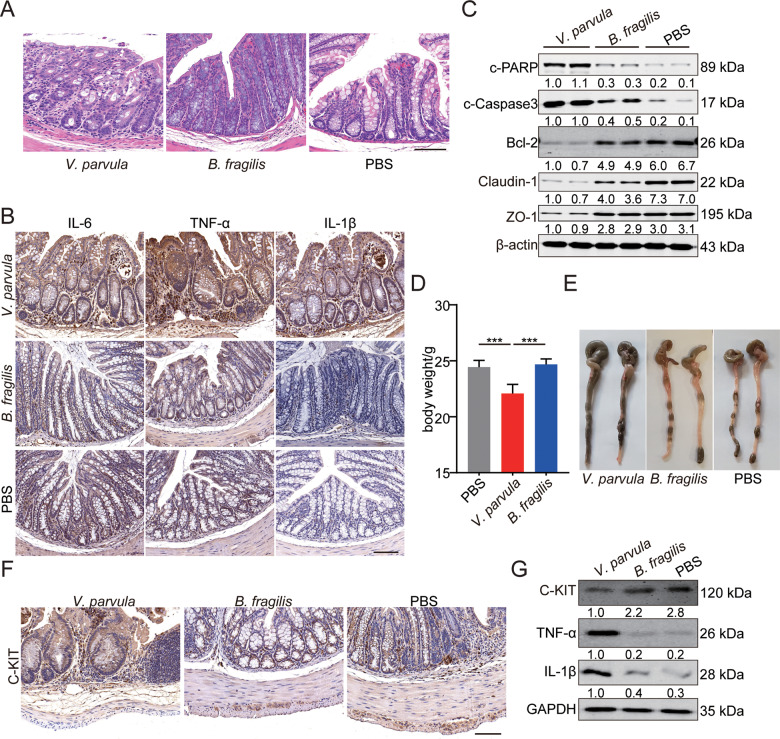
The promoting effects of *V. parvula* overabundance on intestinal inflammation in vivo. **A** Representative photomicrographs showing worse histologic injury, more crypt abscesses and more distribution of immune cells in the distal colon of mice treated with *V. parvula*. Scale bars: 100 μm. **B** Higher levels of inflammatory cytokines (IL-6, IL-1β and TNF-α) in distal colon of mice treated with *V. parvula*. Scale bars: 100 μm. **C** Western blotting assays showing more apoptosis and impaired tight junction induced by *V. parvula* than *B. fragilis* or PBS in distal colon tissues. **D** Body weight of mice treated with *V. parvula* is lighter compared to *B. fragilis* or PBS. **E** Representative images showing more severe intestinal dysmotility and colonic dilatation in mice of *V. parvula* group compared to mice of *B. fragilis* or PBS group. **F** Representative photomicrographs of IHC staining showing less C-KIT^+^ ICC in mice of *V. parvula* group compared to mice of *B. fragilis* or PBS group. Scale bars: 100 μm. **G** Western blotting assays showing more IL-1β and TNF-α and less C-KIT induced by *V. parvula* than *B. fragilis* or PBS in distal colon tissues. All data are expressed as mean ± SD. ***P < 0.001.

As the abundance of *V. parvula* in the intestinal microbiota of HAEC patients was higher than that in the intestinal microbiota of HSCR patients [6], we hypothesized that high levels of *V. parvula* were pro-inflammatory in the intestine. To investigate the effects of the over-proliferation of *V. parvula* on the development of HAEC, germ-free C57BL/6N mice were treated with *V. parvula* by gavage. The abundance of *Bacteroides fragilis* (*B. fragilis*) in gut of HSCR patients was higher than that in HAEC patients, so we employed *B. fragilis* as a negative control in our study. To ensure the quality control in this study, all experiments included were documented to ensure monocolonization only with designated microbe by 16S rDNA sequencing. Besides, feces from different groups were analyzed by deep 16S rDNA sequencing and shown to be pure. Inoculation of *V. parvula* played a pro-inflammatory role and promoted the expression of several inflammatory cytokines (TNF-α, IL-6 and IL-1β) in colon (Fig. 1A, B). Next, we tested several markers of apoptosis and tight junction. *V. parvula* also induced more apoptosis and impaired the tight junction of colonic epithelial cells (Fig. 1C). Besides, treatment with *V. parvula* decreased body weight and affected the mental status of mice (Fig. 1D). The serum inflammatory cytokines didn’t increase significantly, and we didn’t observe other systemic inflammation responses induced by the gavage of *V. parvula*.

In addition, inflated ileocecal part and colonic dilatation were found in mice treated with *V. parvula* (Fig. 1E), indicating that *V. parvula* could induce intestinal dysmotility in colonic tissue. Interstitial cells of Cajal (ICCs) are pacemaker cells that promote intestinal motility [14]. The high level of TNF-α in HAEC patients reduced C-KIT expressions as a biomarker of pacemaker function in ICCs [13]. Consistent with previous study, in the colonic tissues of mice treated with *V. parvula*, we found a reduced number of C-KIT + ICCs compared to those of mice treated with *B. fragilis* (Fig. 1F, G).

Taken together, these results demonstrated that the overabundance of *V. parvula* played a pro-inflammatory role by increasing inflammatory cytokines and intestinal dysmotility in colon.

### The high toxic effects of LPS from *V. parvula* in vivo

**Fig. 2:**
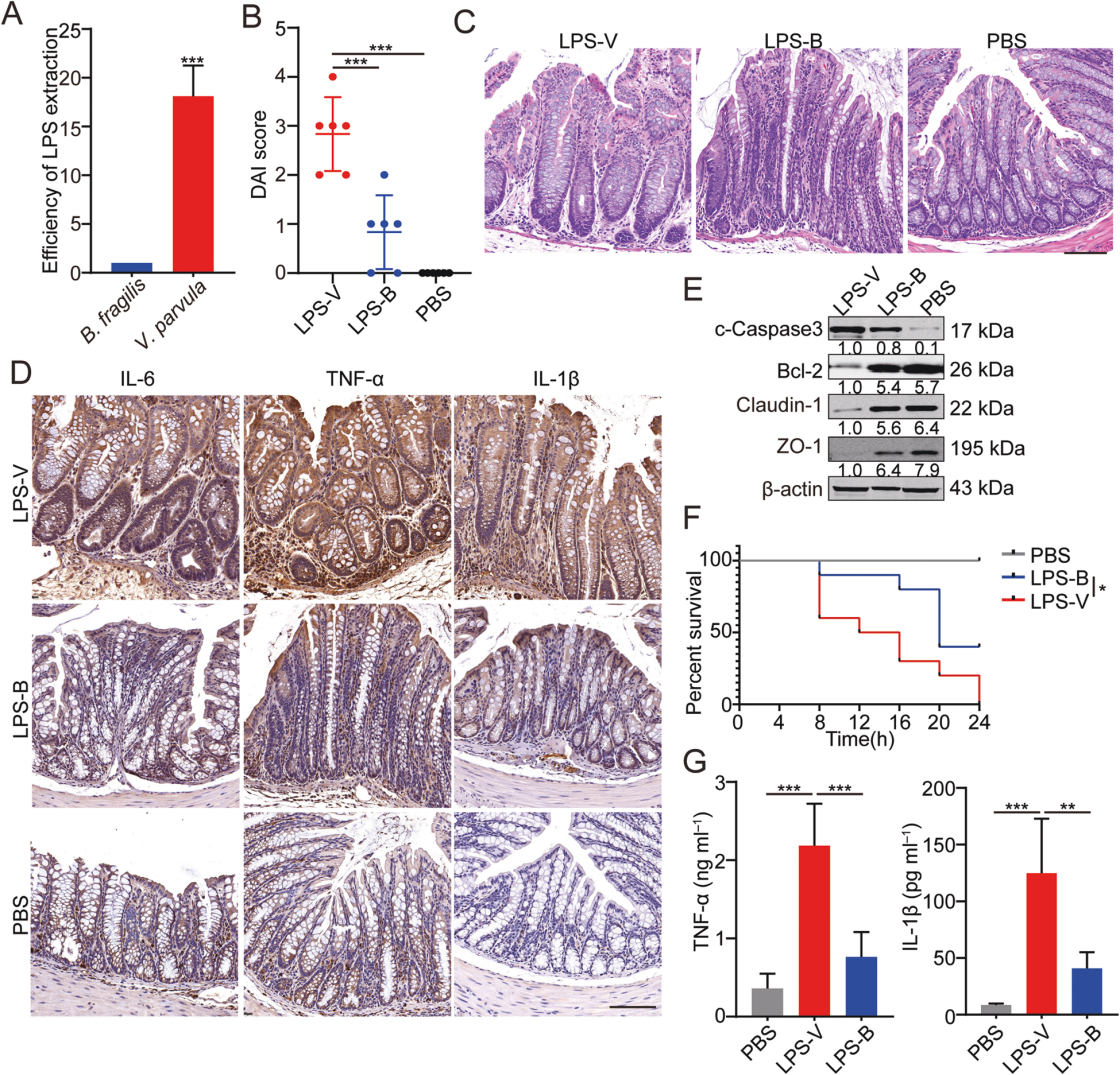
The high toxic effects of LPS from *V. parvula* in vivo. **A** The efficiency of LPS extraction from *V. parvula* and *B. fragilis*. **B** The disease activity index (DAI) of mice in *V. parvula* group is higher than that in *B. fragilis* or PBS group. **C** Representative photomicrographs showing worse histologic injury as well as more crypt abscesses and more distribution of immune cells in the distal colon of mice fed with LPS. Scale bars: 100 μm. **D** Higher levels of inflammatory cytokines (IL-6, IL-1β, and TNF-α) in distal colon of mice treated with LPS-V. **E** Western blotting assays showing more apoptosis and impaired tight junction induced by LPS-V than LPS-B or PBS in distal colon tissues. **F** Survival in mice (*n* = 10 mice/group) treated with LPS-V is shorter compared to mice treated with LPS-B or PBS in an LPS model of sepsis. **G** Serum concentration of pro-inflammatory factors in three groups. All data are expressed as mean ± SD. NS denotes no signification. *P < 0.05; **P < 0.01; ***P < 0.001.

As reported in previous study, postoperative HAEC occurrence is significantly correlated with enteric LPS concentrations [15]. Besides, TLR4 signaling is closely related to ecological imbalance in the intestinal flora and is triggered by LPS from bacteria which results in the upregulation of certain cytokines detected above (Fig. 1B) [16]. Interestingly, the efficiency of LPS extraction from *V. parvula* was 18-fold higher than that from *B. fragilis* (Fig. 2A), indicating that *V. parvula* may induce colitis by releasing LPS.

To confirm the effects of LPS from *V. parvula* (LPS-V) on the development of colitis in vivo, we conducted inflammatory models in mice where inflammation was induced by feeding LPS from *V. parvula* or *B. fragilis* (LPS-B, negative control). According to the condition of diarrhea and bloody stool, we rated the disease activity index (DAI) of mice in each group. It was found that LPS-V caused more serious symptoms related to intestinal inflammation than LPS-B did (Fig. 2B). Next, more damage of epithelial tissue and upregulation of inflammatory cytokines were observed in the colon of mice treated with LPS-V (Fig. 2C, D). LPS-V also induced more apoptosis and impaired the tight junction of colonic epithelial cells compared to LPS-B (Fig. 2E).

Besides, in an LPS model of sepsis in vivo, we found that LPS-V not only increased IL-1β and TNF-α levels but also shortened survival compared to LPS-B did (Fig. 2F, G). These results demonstrated that *V. parvula* promoted the development of intestinal inflammation through releasing LPS in vivo.

### LPS-V impaired the intestinal barrier function of colonic epithelial cells

**Fig. 3:**
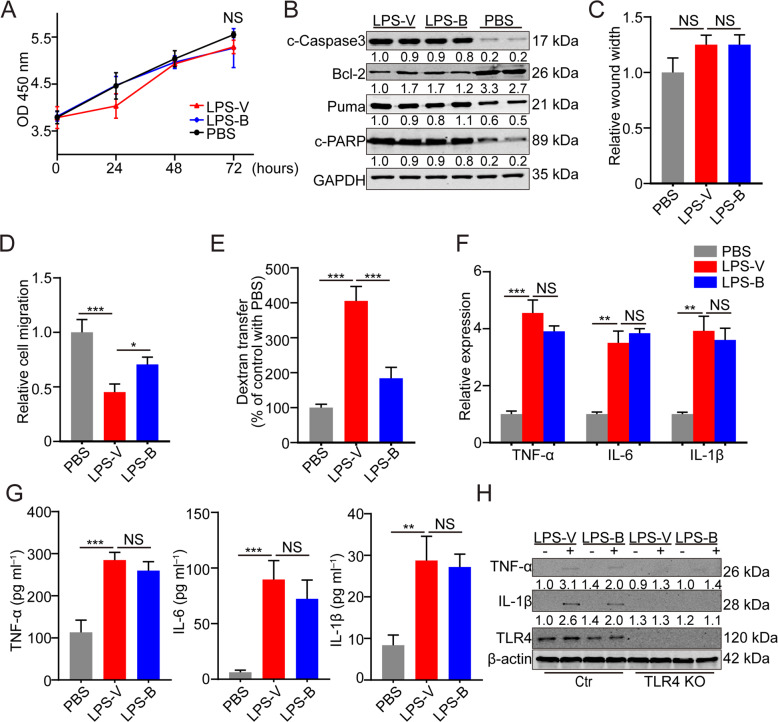
LPS-V impaired the intestinal barrier function of colonic epithelial cells. **A** Cell proliferation was measured by CCK-8 assays in NCM460 cells with treatment of LPS-V, LPS-B or PBS. **B** Western blotting assays showing more apoptosis of NCM460 cells induced by LPS-V or LPS-B than ones induced by PBS. **C** A wound-healing assay was performed to evaluate the migration ability of NCM460 treated with LPS-V, LPS-B or PBS. **D** A transwell migration assay was performed to evaluate the migration ability of NCM460 treated with LPS-V, LPS-B or PBS. **E** A transwell assay using FITC labeled dextran 10 kDa (FD10) was performed to evaluate the paracellular permeability of NCM460 treated with LPS-V, LPS-B or PBS. **F** mRNA expression levels of inflammatory cytokines (IL-6, IL-1β, and TNF-α) in NCM460 treated with LPS-V, LPS-B, or PBS. **G** Supernatant concentration of inflammatory cytokines (IL-6, IL-1β and TNF-α) in NCM460 treated with LPS-V, LPS-B or PBS. **H** Western blotting assays showing that both LPS-V and LPS-B increased the IL-1β and TNF-α expression that was blocked by TLR4 knockout. All data are expressed as mean ± SD. NS denotes no signification. *P < 0.05; **P < 0.01; ***P < 0.001.

The barrier function of colonic epithelial cells is crucial for regulating the interaction between intestinal flora and the internal milieu [17]. To further verify the mechanism of LPS-V induced inflammation reaction in colon, a series of biological experiments were performed. We firstly tested the different effects of LPS derived from *V. parvula* or *B. fragilis* on the normal colonic epithelial cells (NCM460). It was found that LPS from the two species of bacteria had no significant effect on the proliferation and viability of colonic epithelial cells (Fig. 3A). It has been reported that LPS derived from intestinal flora can induce apoptosis of colon epithelial cells [10, 18], so we detected apoptosis markers of colonic epithelial cells treated with different LPS. Both LPS-V and LPS-B could stimulate the apoptosis of colonic epithelial cells, but there was no apparent difference in apoptosis between cells treated by LPS-V and ones treated by LPS-B (Fig. 3B). Then, wound healing and transwell assays were performed to verify the influence of LPS-V on the transfer ability of colonic epithelial cells in vitro. NCM460 cells treated with LPS-V showed no difference in healing speed compared to ones treated with LPS-B (Fig. 3C). However, treatment with LPS-V weakened transfer capability of NCM460 cells compared to LPS-B (Fig. 3D). These data indicated that LPS-V impaired the migration ability but had no influence on proliferation or repair capability of colonic epithelial cells compared to LPS-B did in vitro.

It’s known that colonic epithelial cells express TLR4 on the cytomembrane and are activated by LPS through TLR signaling, resulting in increases of paracellular permeability and expression of inflammatory cytokines [16, 19]. We, therefore, detected the effect of LPS-V treatment on paracellular permeability of colonic epithelial cells using a transwell assay [19]. As shown in Fig. 3E, treatment with LPS-V increased the quantity of FITC labeled dextran that was transferred to the basal compartment, indicating that LPS-V increased the paracellular permeability of NCM460. Next, we used several biochemical experiments and CRISPR/Cas9 system to investigate whether LPS-V affected the expression of inflammatory cytokines in colonic epithelial cells. Both LPS-V and LPS-B induced upregulation and secretion of inflammatory cytokines that were downstream of TLR4 signaling, and there was no difference in these effects of LPS between the two species of bacteria (Fig. 3F, G). The increase of expression of inflammatory cytokines induced by LPS-V or LPS-B were blocked by knockout of TLR4 (Fig. 3H), demonstrating that LPS-V and LPS-B regulated the expression of inflammatory cytokines through TLR4 signaling.

These data indicated that compared with LPS-B, LPS-V caused impaired migration capability as well as increased paracellular permeability of colonic epithelial cells. In addition, LPS-V stimulated the same expression of inflammatory cytokines by TLR signaling as LPS-B did. Together, these results indicated that damaged intestinal barrier function was induced by LPS-V.

### LPS-V promoted the development of intestinal inflammation through activating macrophages

**Fig. 4:**
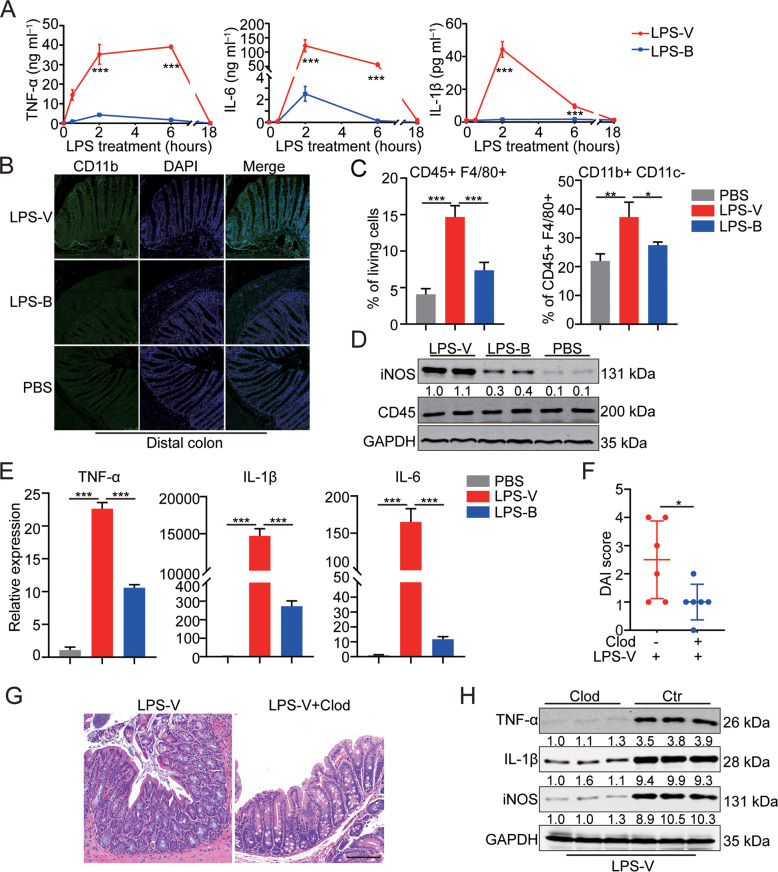
LPS-V promoted the development of intestinal inflammation through activating macrophages. **A** Supernatant concentration of inflammatory cytokines (IL-6, IL-1β and TNF-α) in BMDMs treated with LPS-V or LPS-B at indicated time points. **B** Representative photomicrographs of IF staining showing more CD11b + macrophages in mice of LPS-V group compared to LPS-B or PBS group. Scale bars: 100 μm. **C** Flow cytometric histograms indicating the detection of pro-inflammatory macrophages in distal colon of mice treated with LPS-V, LPS-B, or PBS. **D** Western blotting assays showing that LPS-V increased iNOS level in distal colon. **E** mRNA expression levels of inflammatory cytokines (IL-6, IL-1β, and TNF-α) in human macrophages treated with LPS-V, LPS-B, or PBS. **F** The disease activity index (DAI) of mice indicating macrophage depletion using Clod treatment alleviated the effects of LPS-V. **G** Representative photomicrographs showing macrophage depletion using Clod treatment alleviated the effects of LPS-V. on colon tissue. Scale bars: 100 μm. **H** Western blotting assays showing macrophage depletion using Clod treatment decreased IL-1β, TNF-α and iNOS level in distal colon. All data are expressed as mean ± SD. *P < 0.05; **P < 0.01; ***P < 0.001.

Macrophages originating from monocytes play important roles in immune system of vertebrates, including innate immunity and cell-mediated immunity [20]. Besides, macrophages are activated by LPS to function through the expression and secretion of pro-inflammatory cytokines and sequentially promote an anti-inflammatory phenotype to limit intestinal tissue damage and promote tissue repair [21]. We, therefore, conjectured that dysregulation of macrophages caused by LPS-V may aggravate HAEC. Bone marrow-derived macrophages (BMDMs) stimulated by LPS-V produced much higher and more persistent inflammatory cytokines compared to LPS-B did (Figs. 4A and S1A). In the inflammatory models by feeding LPS, we found that M1 macrophages increased significantly in the group of LPS-V but increased slightly in the group of LPS-B (Figs. 4B–D and S1B). LPS-V can also induce more expression of pro-inflammatory cytokines than LPS-B did in human macrophages (Fig. 4E).

To determine the role of macrophages in enterocolitis induced by LPS-V, macrophage depletion by Clodronate-containing Liposomes (Clod) in vivo were conducted before and during treatment with LPS-V [22]. As shown in Fig. 4F, G, macrophage depletion alleviated symptoms of colitis induced by LPS-V, and the level of inflammatory cytokines in the group of macrophage depletion was much lower than that in the control group (Fig. 4H). These results demonstrated that LPS-V promotes the development of intestinal inflammation by activating macrophages.

### LPS-V activated macrophages through TLR4 signaling

**Fig. 5:**
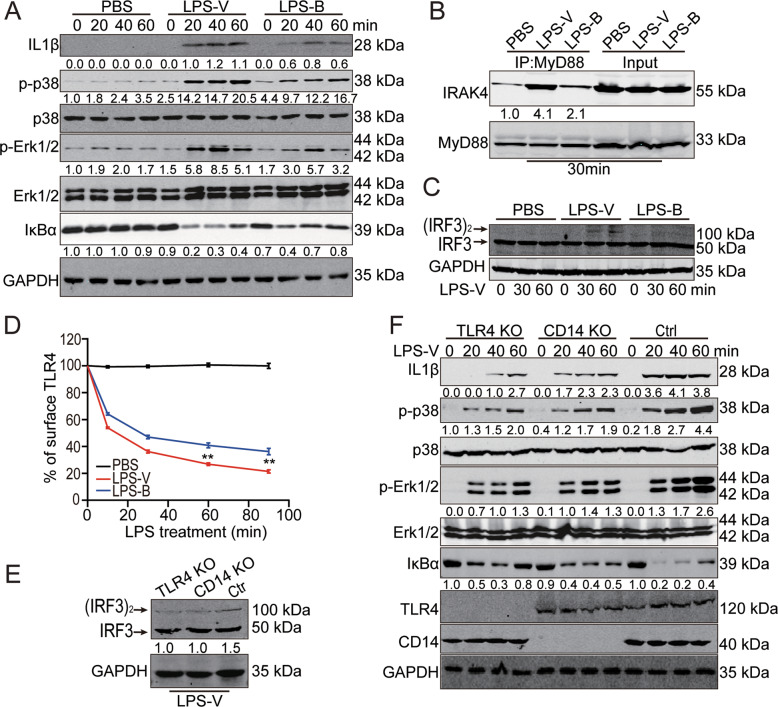
LPS-V activated macrophages through TLR4 signaling. **A** Western blotting assays showing IL-1β and TNF-α as well as MyD88-dependent changes in IκBα levels, p-p38, and p-ERK1/2 at indicated time points of treatment with LPS (1 μg/ml) in BMDMs. **B** IP and Western blot analysis of the complex of MyD88 and IRAK4 30 min post treatment with LPS (1 μg/ml) in BMDMs. **C** Western blotting assays using Native-PAGE electrophoresis indicating active (dimerized) IRF3 at indicated time points of treatment with LPS (1 μg/ml) in BMDMs. **D** Mean fluorescence intensity (MFI) of TLR4 receptor staining at each time point of treatment with LPS (1 μg/ml) in BMDMs. Data are expressed as mean ± SD. **P < 0.01. **E** TLR4 or CD14 KO decreased active (dimerized) IRF3 in macrophages differed from THP1 treated with LPS-V. **F** Western blotting assays showing that TLR4 or CD14 KO decreased IL-1β and TNF-α as well as MyD88-dependent IκBα levels, p-p38, and p-ERK1/2 at indicated time points of treatment with LPS (1 μg/ml) in macrophages differed from THP1.

M1 macrophages (also called inflammatory macrophages) play a key role in the innate and adaptive immune responses against pathogens [23]. However, the over-activation of these macrophages results in the development and progression of various inflammatory diseases, including a series of intestinal diseases. LPS from pathogens can trigger functional polarization of the activated macrophages by binding TLR4 on the surface of macrophages [16]. Therefore, we next tested whether LPS-V activated macrophages and increased inflammatory cytokines through TLR signaling. We found that treatment with LPS-V induced more intense signal transduction and higher levels of inflammatory cytokines in macrophages (Fig. 5A and Fig. S1C), including TRIF-mediated signaling pathway and TIRAP/MyD88-mediated signaling pathway (Fig. 5A–C).

The endocytosis of TLR4, which are responsible for detecting microbial products and triggering innate and adaptive immunity, has emerged as a critical control step in the process of signal transduction. To identify the effects of LPS-V on endocytosis of TLR4, we employed a highly sensitive flow cytometry assay to detect endogenous TLR4, using the loss of cell surface staining as an event of TLR4 endocytosis as reported [24]. Treatment with LPS-V reduced TLR4 expression on the surface of BMDMs (Fig. 5D), indicating that LPS-V enhanced endocytosis of LPS-V and TLR4 signal transduction. To further confirm our hypothesis that LPS-V induced dysregulation of macrophages by TLR4, macrophages differed from THP1 (human acute monocytic leukemia) which was a classic model that was drawn into our studies [25]. CD14 is involved in the endocytosis of TLR4, and defect of CD14 impaired the endocytosis of TLR4 and the activation of TLR4 downstream pathway [24]. We conducted THP1 cell lines with TLR4 or CD14 knockout and then induced the differentiation of THP1 into macrophages with stimulation of PMA (Fig. S1D). TLR4 or CD14 knockout impaired TLR4 signaling transduction and decreased the expression of inflammatory cytokines in macrophages when treated by LPS-V (Fig. 5E, F), indicating that TLR4 signaling played an important role in the pro-inflammatory effect of LPS-V on macrophages.

### OI inhibited the development of LPS-V induced intestinal inflammation by repressing macrophage function

**Fig. 6:**
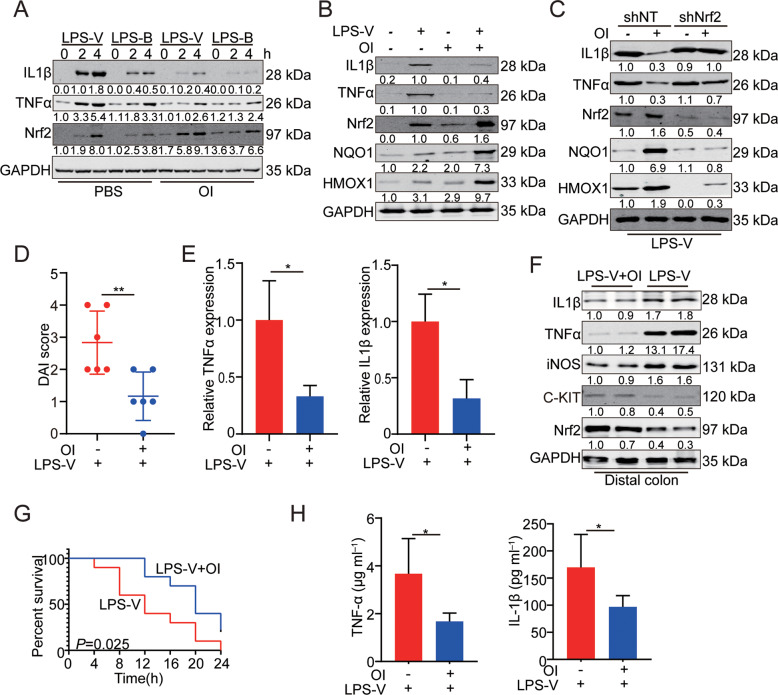
OI inhibited the development of LPS-V induced intestinal inflammation by repressing macrophage function. **A** OI treatment decreased IL-1β and TNF-α but increased Nrf2 levels at indicated time points of treatment with LPS (1 μg/ml) in BMDMs. **B** OI treatment decreased IL-1β and TNF-α but increased Nrf2 levels and Nrf2 target gene (NQO1 and HMOX1) expression 30 min post treatment with LPS-V (1 μg/ml) in human macrophages. **C** Nrf2 KD alleviated the decreasing effects of OI on IL-1β and TNF-α by repressing Nrf2 target gene (NQO1 and HMOX1) expression in BMDMs treated LPS-V (1 μg/ml). **D** The DAI score of mice indicating OI treatment alleviated the effects of LPS-V. **E** mRNA expression levels of inflammatory cytokines (IL-1β and TNF-α) were decreased by OI. **F** Western blotting assays showing OI treatment decreased inflammatory cytokines and iNOS levels but increased the expression of Nrf2 and C-KIT in distal colon. **G** OI treatment prolonged survival of mice (*n* = 10 mice/group) treated with LPS-V in an LPS model of sepsis. **H** OI treatment decreased serum concentration of IL-1β and TNF-α in an LPS model of sepsis. All data are expressed as mean ± SD. *P < 0.05; **P < 0.01.

Itaconic acid (itaconate), a metabolite synthesized by IRG1 in mitochondrion, has recently emerged as an inflammatory regulator [26]. Macrophages are activated by LPS to synthesize more itaconate in mitochondrion, which sequentially limits inflammation. KEAP1 interacts with the anti-inflammatory transcription factor Nrf2 and promotes the degradation of Nrf2. Itaconate plays an anti-inflammatory role via alkylation of KEAP1 cysteine residues. The alkylation of KEAP1 cysteine impairs the degradation of Nrf2 (Fig. S2A), which accumulates and migrates to the nucleus to activate a transcriptional anti-oxidant and anti-inflammatory program as previously reported [27]. Treatment with 4-octyl itaconate (OI), a cell-permeable itaconic acid derivative, neutralized the effects of LPS-V on the expression of inflammatory cytokines by activating Nrf2 (Fig. 6A, B). OI allowed newly synthesized Nrf2 to accumulate and migrate to the nucleus (Fig. S2B), so a transcriptional anti-oxidant and anti-inflammatory program were triggered. We also found that HMOX1 and NQO1, anti-inflammatory downstream of Nrf2 [27], were upregulated by OI (Fig. 6B). Nrf2 knockdown significantly enhanced inflammatory response in macrophages and impaired the reduction of IL-1β and TNF-α induced by OI (Fig. 6C). Our data indicated that OI counteracted the effects of LPS-V through activating Nrf2 and then reduced pro-inflammatory response of macrophages in vitro.

OI ameliorated the condition of the colon and reduced the expression of TNF-α and IL-1β in mice of inflammatory models induced by feeding LPS-V (Fig. 6D–F). OI also increased the expression of C-KIT indicating a restored ICC phenotype (Fig. 6F). Pre-treatment with OI also decreased the circulatory concentration of pro-inflammatory cytokines induced by LPS-V and prolonged survival in a model of sepsis in vivo (Fig. 6G, H).

These results suggest that itaconic acid or OI represses LPS-V-induced inflammation in macrophages by stabilizing Nrf2 and upregulating the anti-inflammatory downstream of Nrf2, which provides us with therapeutic opportunities to use itaconic acid or OI to treat LPS-V-induced HAEC.

## Discussion

Maintaining the homeostasis of microbial communities in the human gastrointestinal tract is significant for health, and the alteration of intestinal flora is associated with numerous health disorders [28, 29].

In previous studies, abnormal changes in intestinal flora were found in HAEC patients. We also found a lower diversity of gut microflora in different intestinal segments of HAEC patients compared with that of HSCR patients. In our previous study, the abundance of *Veroniella* in the intestinal tract of HAEC patients was found to be abnormally high at the species level, where *V. parvula* was the main species [6]. The transplantation of *Veillonella spp*. into germ-free C57BL/6N mice by gavage of a broth-grown single-bacterial strain could affect the intestinal immune system and reduce the expression of IL-22 in colon [30]. The high expression of IL-22 might inhibit the development of inflammatory bowel disease (IBD) [31]. Besides, the abundance of *Veillonella* increased in patients with active status of primary sclerosing cholangitis (PSC) and ulcerative colitis (UC) [9]. In our single-bacterial strain transplantation models, we found that *V. parvula* could cause colitis-like symptoms including infiltration of immune cells, intestinal dysmotility, and intestinal mucosal damage (Fig. 1). However, the molecular mechanism of colitis caused by abnormal intestinal flora remains unclear.

Intestinal flora can affect intestinal tissues through a variety of components including soluble peptides or toxins, cellular structural components, and metabolites. Among these, LPS can increase the paracellular permeability of colonic epithelial cells by affecting tight junctions. The function of LPS released by different bacteria and the response of different receptor molecules to LPS are different [32]. It has been reported that some modifications of LPS can significantly enhance the activation of the molecule to Toll-like receptor 4-myeloid differentiation factor 2 (TLR4-MD2) complexes on immune cells and produce more pro-inflammatory factors [33]. In this study, we found that the extraction efficiency of LPS from *V. parvula* was much higher than that of *B. fragilis* (negative control). It is also found that LPS from *V. parvula* increased the paracellular permeability of colonic epithelial cells and promoted colitis-like symptoms by activating macrophages. The activated M1 macrophages in colonic tissues produce a large amount of pro-inflammatory factors including TNF-α, IL-6, and IL-1β. The pacemaker function of Interstitial cells of Cajal (ICCs) was sequentially impaired by TNF-α from M1 macrophages, and thus the intestinal motility was damaged [13]. However, models we used in this study did not fully simulate the HAEC condition. It is hard to obtain infant mice with HSCR. Our data could only indicate the pro-inflammatory role of *V. parvula* by releasing LPS, but couldn’t demonstrate the effects of *V. parvula* on HAEC. This is a limitation of our study. In order to further study the pathogenesis and drug screening, we need to establish a mouse model that simulates the HAEC condition in future researches. We proved that LPS-V promoted the development of HAEC through macrophage-related inflammation. Macrophage polarization occurred during LPS-V processing, where M1 macrophages infiltration increased dramatically in colonic tissues. The LPS-V treatment can induce more intense and persistent production of inflammatory factors in macrophages than the LPS-B treatment does. TLR4 is the main receptor of LPS and is widely distributed in various cells and tissues. TLR4 or CD14 knockout could significantly reduce the LPS-V-induced production of pro-inflammatory factors in macrophages, suggesting that LPS-V-induced macrophage-related inflammation through TLR4 pathway. It has been reported that the concentration of serum LPS was positively correlated with the occurrence of HAEC [15]. LPS entering the blood would cause systemic diseases such as age-related atrial fibrillation. LPS-induced model of sepsis in vivo showed that LPS-V-induced higher levels of serum inflammatory factors and shortened the survival of mice compared to LPS-B (Fig. 2). The TNF-α produced by M1 macrophages can damage the pacemaker function of ICCs, resulting in intestinal dysmotility [13]. Therefore, the overgrowth of *V. parvula* in HAEC patients could damage intestinal motility through inflammatory responses. The intestinal dysmotility could lead to fecal accumulation and excessive bacteria growth, which could further aggravate the alteration of intestinal flora.

Itaconic acid is synthesized by IRG1 protein in mitochondria and is able to regulate inflammatory response [34]. 4-octyl itaconate (OI), a cell-permeable itaconate derivative, can improve the stability of Nrf2 by inhibiting the binding of KEAP1 and Nrf2 [21]. Nrf2 can enter the nucleus and activate a transcriptional anti-oxidant and anti-inflammatory program. In this study, OI treatment increased the expression of Nrf2 in colonic epithelial cells and macrophages, activated the expression of downstream anti-oxidant and anti-inflammatory genes, and inhibited the pro-inflammatory factors produced by macrophages. In the mouse model, OI treatment could significantly alleviate the colitis-like symptoms caused by LPS-V, including the reduction of pro-inflammatory factors and the promotion of intestinal motility (Fig. 6).

## Conclusion

**Fig. 7:**
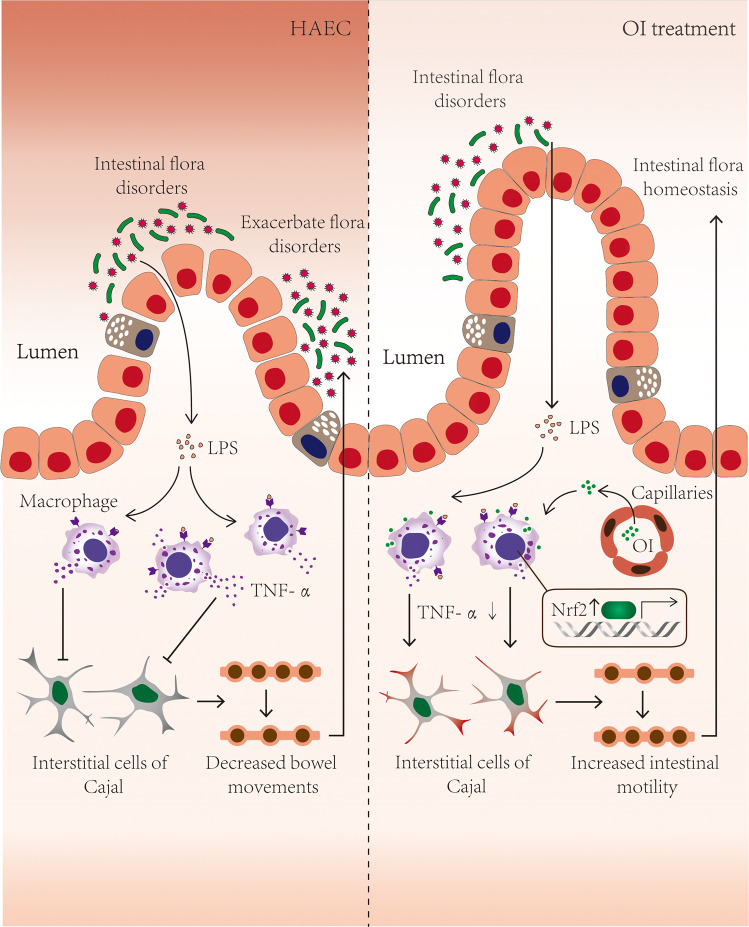
The vicious cycle involving intestinal flora, LPS, macrophages, TNF-α, and ICC. Increased LPS from *V. parvula* resulting from intestinal flora dysbiosis could significantly enhance the paracellular permeability of colonic epithelial cells and activate macrophages through TLR4 pathway. TNF-α from polarized macrophages impaired pacemaker function of ICCs and then inhibited intestinal motility. Intestinal dysmotility aggravated the intestinal flora dysbiosis and promoted the development of HAEC (left). OI could break the vicious cycle by inhibiting the activation of macrophages through activating Nrf2, and then promote intestinal flora homeostasis.

We found that LPS from *V. parvula* can significantly increase the paracellular permeability of colonic epithelial cells, and then activate macrophages through TLR4 pathway. The activated macrophages became polarized and produced pro-inflammatory factors. Then the pacemaker function of ICCs was inhibited and intestinal dysmotility was aggravated. It was thus a vicious cycle, where the macrophage-related inflammation caused by *V. parvula* through LPS damaged the intestinal motility, which further aggravated the intestinal flora dysbiosis and promoted the development of HAEC. By activating Nrf2, OI could break the vicious cycle by inhibiting the activation of macrophages induced by LPS-V (Fig. 7). Therefore, treatment with OI targeting macrophages could be a potential therapeutic strategy for HAEC patients with intestinal flora dysbiosis.

## Methods

### Cell lines

The normal colonic immortalized epithelial cell line NCM460 (purchased from INCELL), THP-1 (ATCC® TIB-202), and HEK293T (ATCC® CRL11268) were cultured in RPMI 1640 or Dulbecco’s Modified Eagle Medium (DMEM) with 10% fetal bovine serum(FBS) and incubated at 37 °C with 5% CO_2_.

### Mice

C57BL/6 mice were from Shanghai SLAC Laboratory Animal Co. Ltd (Shanghai, China). All procedures for mice were performed in accordance with the relevant laws and guidelines of the Institutional Animal Care and Use Committee (IACUC) of Shanghai Jiao Tong University.

### Microbe strains

*V. parvula* and *B. fragilis* were purchased from ATCC and were cultured in Wilkins-Chalgren anaerobe broth (thermo fisher Scientific) in anaerobic jar (80% N_2_, 10% H_2_, 10% CO_2_) at 37 °C.

### Microbiota transplantation

Microbiota transplantation was performed as described previously [30]. Germ-free C57BL/6 mice from Cyagen Biosciences were fed in flexible film isolators, being ventilated via filtered air at 22 ± 1 °C with a 12-h light/dark cycle. Germ-free C57BL/6 mice were randomly assigned to treatment groups before experiments. Germ-free mice were treated with capsules containing *V. parvula* or *B. fragilis* three times for three consecutive days by intragastric administration. Euthanasia of mice were performed at the end of experiments. All procedures for mice were performed in accordance with the relevant laws and guidelines of the Institutional Animal Care and Use Committee (IACUC) of Shanghai Jiao Tong University.

### LPS feeding models

LPS feeding models were constructed according to the previous study [32]. Briefly, C57BL/6 mice were fed with 160 μg LPS from *V. parvula* or *B. fragilis* once by oral gavage and received 100 μg/ml LPS in drinking water for 2 weeks. Euthanasia of mice were performed at the end of experiments. All procedures for mice were performed in accordance with the relevant laws and guidelines of the Institutional Animal Care and Use Committee (IACUC) of Shanghai Jiao Tong University.

### Colon histopathology and immunohistochemistry (IHC) staining

Mouse colon tissues were cut and fixed in 4% paraformaldehyde (PFA) for 24 h. Then fixed tissues were embedded in paraffin, sectioned, and stained using Hematoxylin and Eosin Staining Kit (Beyotime Biotechnology). Immunohistochemistry (IHC) was performed as we described previously [35]. Briefly, tissue sections were incubated by appropriate primary antibodies overnight at 4 °C, followed by incubation with GTVisionTM III Detection System/Mo & Rb/including DAB (gene tech, Shanghai). The cell nucleus was stained with hematoxylin (Sigma-Aldrich). The stained sections were scanned with Leica versa 8. Anti-IL1β antibody was purchased from Cell Signaling Technology. Anti-c-Kit antibody was obtained from Abcam. Anti-TNFα antibody were purchased from Santa Cruz biotechnology. Anti-IL6 antibody were purchased from Servicebio.

### Immunoblotting (IB) and immunoprecipitation (IP)

Immunoblotting (IB) and immunoprecipitation (IP) were performed as we described previously [36]. Briefly, cells or tissues were lysed in RIPA buffer (20 mM Tris-HCl, pH 7.5, 150 mM NaCl, 1 mM EDTA, 2 mM Na3VO4, 5 mM NaF, 1% Triton X-100) with Protease inhibitor cocktail (invitrogen). For immunoprecipitation, the protein lysates were incubated with appropriate antibodies, captured by protein G plus-agarose (Santa Cruz), and eluted by SDS loading buffer. The proteins were resolved in SDS-PAGE or Native-PAGE electrophoresis, transferred to PVDF membranes, and detected by the appropriate antibodies. After incubation with appropriate secondary antibodies (CST), the membrane was visualized and analyzed by an imaging system (Bio-Rad Laboratories).

### mRNA expression analyses

Total RNA was extracted using MolPure® Cell/Tissue Total RNA Kit (YEASEN) and quantified using a Nanodrop 2000 UV-visible spectrophotometer. Complementary DNA (cDNA) was synthesized from 1 μg of total RNA using the PrimeScript RT–PCR Kit (Takara) according to the manufacturer’s instructions. Quantitative real-time PCR was performed on a CFX Connect^TM^ Real-Time System (Bio-Rad Laboratories) with Hieff® qPCR SYBR Green Master Mix (YEASEN) following the manufacturer’s protocol. Fold changes were calculated using the ΔΔCt method with mouse or human GAPDH as an endogenous control for mRNA expression. The qPCR primers used for validation are listed in Supplementary Table S1.

### Cell viability

Cell viability was detected by Cell Counting Kit 8(YEASEN) following the manufacturer’s instructions. Cells were plated into 96-well plates at 0 h. Cells were treated with CCK-8 and deteted by SYNERGY2 microplate reader (BioTek Instruments) at 450 nm. OD value indicated the cell viability. The experiment was performed in triplicate.

### Wound healing

Wound healing assay was performed as we described previously [35]. Cells were plated into six-well plates at 0 h. we gently scratched the monolayer with a pipette tip to create a mechanical wound when the cells reached confluence. A microscope was used for imaging at 0 and 48 h. And we calculating the gap size in each field. The experiment was performed in triplicate.

### Transwell assays

Transwell assays were performed using 24-well Boyden chamber transwell inserts (BD Biosciences, USA) as described previously [35]. Cells (1 × 10^5^ in 200 μL of DMEM without FBS) were added to the upper chambers. The lower chamber of each well contained 600 μL of DMEM with 15% FBS. After incubation in an incubator at 37 °C in 5% CO_2_ for 24 h, cells that migrated to the bottom of the upper chamber membrane were stained with crystal violet and counted. The experiment was performed in triplicate.

### CRISPR/Cas9 knockout

Guide RNA sequences targeting human TLR4 (ATTCTCCCAGAACCAAACGA, ATGCCCCATCTTCAATTGTC) or CD14(CGGTGGCGCGCAGCGAGTTG) were designed using the MIT online tool (http://crispr.mit.edu) and cloned into a lentiCRISPRv2 vector as previously described [36]. THP1 cells were infected with viral supernatants from HEK293T cells that were transfected by certain plasmids and selected with puromycin. The knockout efficiency of TLR4 or CD14 was confirmed by immunoblotting.

### Generation of BMDMs

Generation of BMDMs was performed according to previous study [21]. Bone marrow (BM) cells were isolated from femurs of 8–12 weeks old male mice. BM cells were treated with ACK lysis buffer for 5 min to remove red blood cells, centrifuged for 5 min at 300 g, and resuspended in BMDM medium (DMEM supplemented with 10% v/v heat-inactivated FBS, 2 mM L-glutamine, 100 units/mL, 100 μg/mL penicillin/streptomycin, 0.5 mM sodium pyruvate and 100 ng/ml M-CSF). Cells were maintained at 37 °C in a humidified 5% CO2 incubator. Medium was changed at day 4 of differentiation, and then Bone Marrow-Derived Macrophages (BMDMs) were obtained at day 6 of differentiation and replated for experiments.

### Isolation of human PBMCs

Human peripheral blood mononuclear cells (PBMCs) were isolated from human blood using Ficoll (GE). Whole blood (15 ml) mixed with RPMI1640 (15 ml) was layered on 10 ml Ficoll and spun for 20 min at 2000 rpm with no brake on. The PBMCs were isolated from the middle layer. PBMCs were counted and then maintained in RPMI 1640 supplemented with 10% FBS, 2 mM L-glutamine, and 1% penicillin/streptomycin solution.

### Generation of human macrophages

Human PBMCs were sorted by magnetic-activated cell sorting (MACS) CD14 beads following the manufacturer’s instruction and then plated at 0.5 × 10^6^ cells/ml with RPMI 1640 supplemented with 10% FBS, 2 mM L-glutamine, 1% penicillin/streptomycin solution and 100 ng/ml M-CSF. After 5 days of differentiation, human macrophages were counted and replated at 0.5 × 10^6^ cells/ml for further experiments.

### LPS extraction

The extraction of lipopolysaccharide (LPS) was performed using LPS Extraction Kit (iNtRON) following the manufacturer’s instructions. The concentration measurement of LPS were carried out by EC Endotoxin Test Kit (BIOENDO, Xiamen, China) according to the manufacturer’s instruction.

### Paracellular permeability

Paracellular permeability assay was performed according to previous study [19]. Briefly, NCM460 were plated in apical compartment and proliferated until cells covered ~100% of area. Then, FITC labeled dextran 10 kDa (FD10) was added into the apical compartment (1 mg/ml) and its amount in the basal compartment was measured using a SYNERGY2 microplate reader (BioTek Instruments). The amount of FITC labeled dextran in the basal compartment represents the paracellular permeability of colonic epithelial cells in different groups.

### Immunofluorescence (IF)

IF was performed as we described previously [37]. Briefly, tissue sections were fixed in 4% paraformaldehyde for 30 min, permeabilized with 0.5% Triton X-100 in PBS for 30 min and blocked with 10% donkey serum in PBS for 2 h at room temperature. After incubation with Alexa 488 labeled anti-CD11b antibody overnight at 4 °C, cells were washed with PBS for three times the next day and incubated with secondary antibodies conjugated with Alexa 488. DAPI was used as nuclear staining. Images were obtained and analyzed using Leica SP8.

### Flow cytometry

Mouse colon tissues were cut and cleaned to remove fat tissue and blood vessels. Then epithelial cells and mucus were removed by 40 min incubation with HBSS (without Ca2^+^ and Mg2^+^) containing 5% FBS, 2 mM EDTA, and 0.15 mg/ml (1 mM) DTT (Sigma) at 37 °C shaking at 250 r.p.m. Colon pieces were then digested in PBS containing 5% FBS, 1 mg/ml Collagenase VIII (Sigma), and 0.1 mg/ml DNase I (Roche) for 40 min at 37 °C shaking at 250 r.p.m. The single cells were isolated from digested cell suspension using 100 μm cell strainers and stained with appropriate antibodies for 30 min at room temperature. Anti-CD11b, anti-F4/80, and anti-CD11c, anti-CD45 antibodies were purchased from Invitrogen.

Fluorescence was detected using a BD FACS Canto flow cytometer (Becton Dickinson, Mountain View, CA, USA) and analyzed using FlowJo software (version 7.6.1). The experiment was performed in triplicate.

### LPS-induced model of sepsis in vivo

LPS-induced model of sepsis was constructed according to previous study [21]. Mice were treated with LPS stimulation by intraperitoneal injection. Then mice were euthanized in a CO_2_ chamber, blood samples were collected at different time points and serum was isolated. Then cytokines in serum were measured by V-PLEX Proinflammatory Panel 1 Mouse kit (Meso Scale Discovery) according to the manufacturer’s protocol. The survival status of mice in different groups was recorded.

### Macrophage depletion in vivo

Macrophage depletion was performed as described previously [22]. Mice were treated intraperitoneally with 200 μL of Clod (5 mg/mL) (FormuMax Scientific Inc., Sunnyvale, USA) 4 day prior to LPS stimulation and every 2 days during experiment in vivo.

### shRNA knockdown

The sequences of all small hairpin RNA (shRNA) targeting Nrf2 (GCTCCTACTGTGATGTGAAAT) or non-target (TTCTCCGAACGTGTCACGT) were synthesized and cloned into a pll3.7 vector. BMDM were transfected with the indicated shRNA twice at 24 h intervals using jetOPTIMUS® (Polyplus-transfection) before LPS treatment. The knockdown efficiency of Nrf2 was confirmed by immunoblotting.

### Quantitative and statistical analysis

Student’s *t* test and ANOVA were used to analyze the data from two groups and multiple groups in this study. Statistical significance was described by *p* value in the methods and figure legends. *P < 0.05; **P < 0.01; ***P < 0.001.

## Supplementary information

S1

S2

Supplementary legends

table S1

## Data Availability

The datasets used and analyzed during the current study are available from the corresponding author on reasonable request.
