# Supplementary figures and images for: Overabundance of Veillonella parvula promotes intestinal inflammation by activating macrophages via LPS-TLR4 pathway
Source: Cell Death Discov. 2022 May 6;8:251. doi: 10.1038/s41420-022-01015-3 (PMC9076897; doi:10.1038/s41420-022-01015-3)

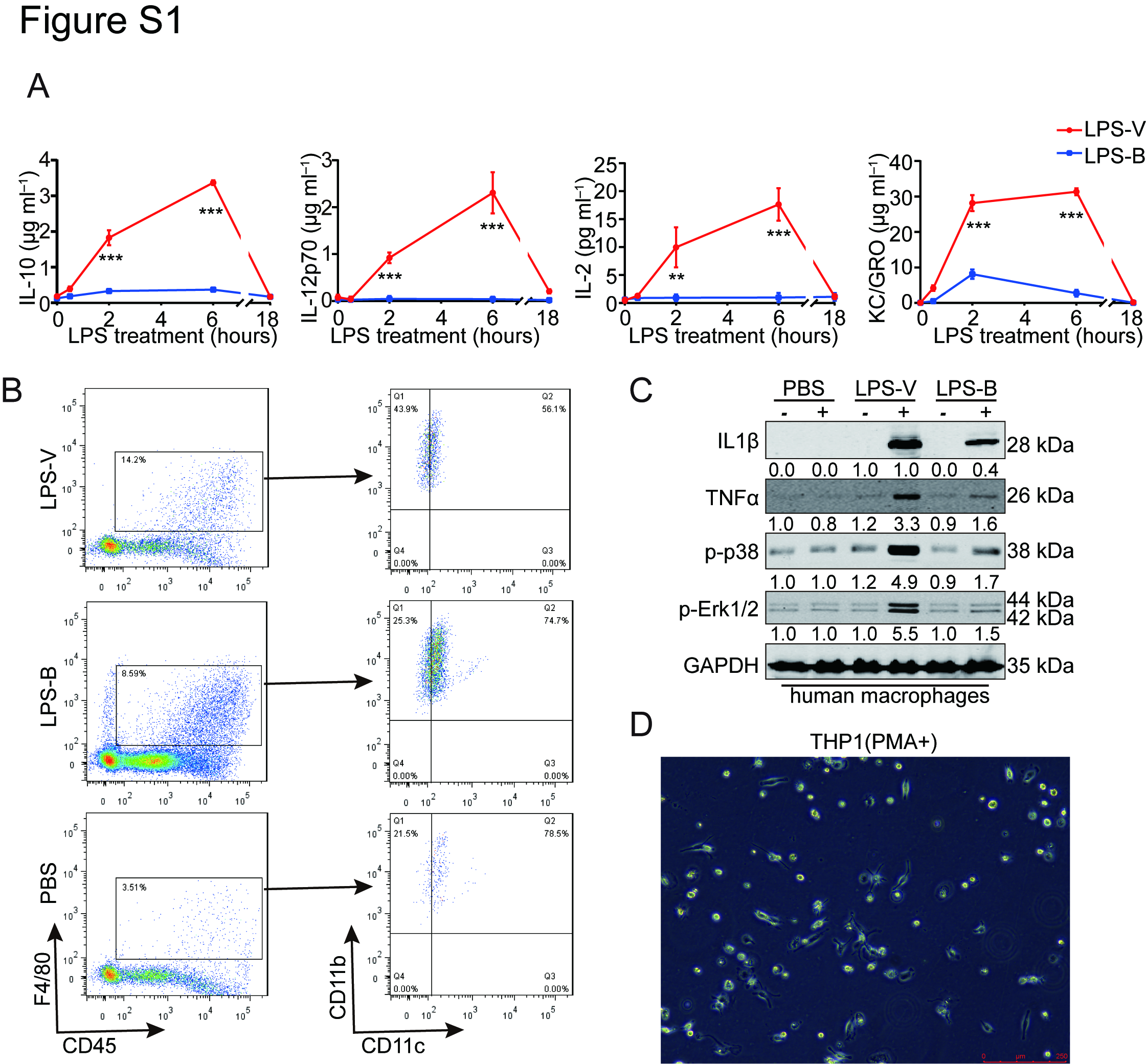

Supplement: Supplementary file 1 — S1 [file 41420_2022_1015_MOESM1_ESM.tif]

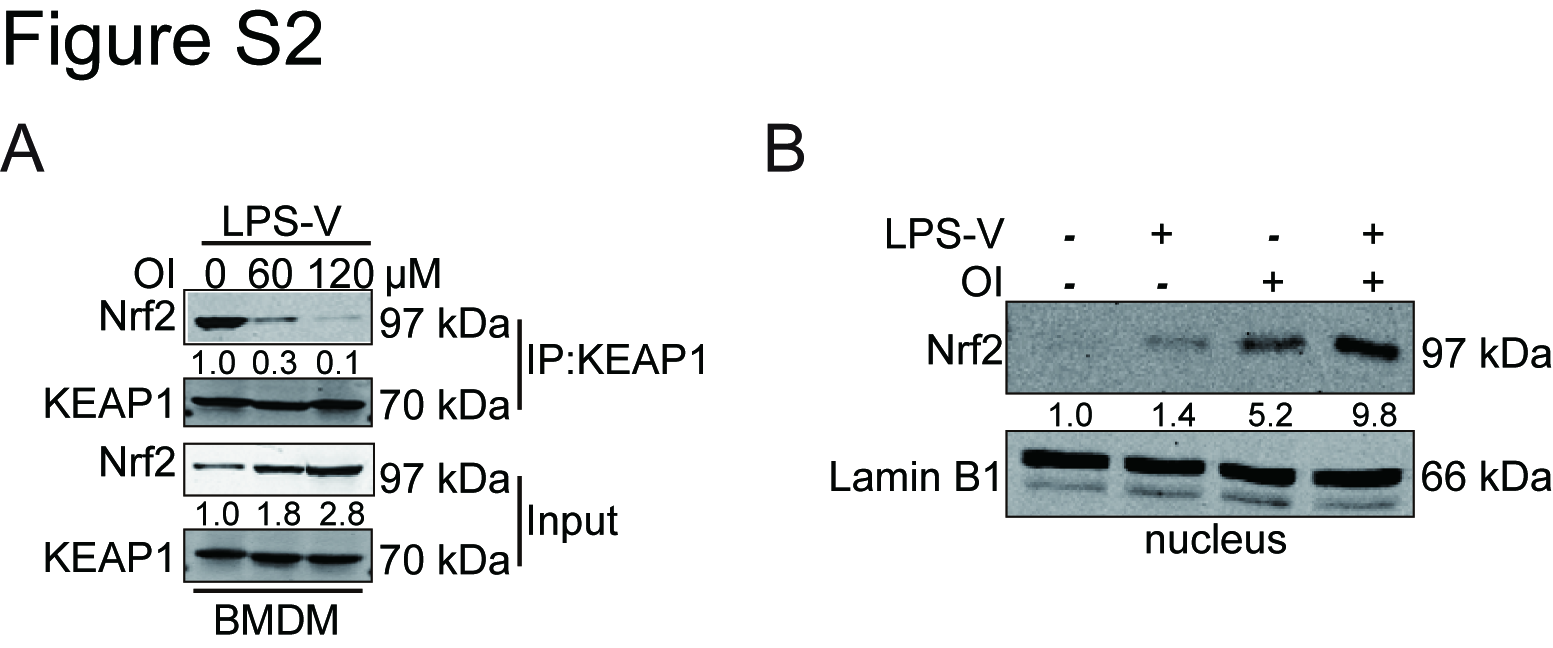

Supplement: Supplementary file 2 — S2 [file 41420_2022_1015_MOESM2_ESM.tif]
