# Supplementary material for: Overabundance of Veillonella parvula promotes intestinal inflammation by activating macrophages via LPS-TLR4 pathway
Source: Cell Death Discov. 2022 May 6;8:251. doi: 10.1038/s41420-022-01015-3 (PMC9076897; doi:10.1038/s41420-022-01015-3)
Supplement: Supplementary file 3 — Supplementary legends [file 41420_2022_1015_MOESM3_ESM.docx]

**Figure S1**

1. Supernatant concentration of inflammatory cytokines (IL-2, IL-10, IL-12p70 and KC/GRO) in BMDMs treated with LPS-V or LPS-B at indicated time points. All data are expressed as mean ± SD. ** P <0.01; *** *P* < 0.001.
2. Representative pictures of Figure 4C.
3. Western blotting assays showing IL-1β and TNF-α as well as p-p38 and p-ERK1/2 at indicated time points of treatment with LPS (1μg/ml) in human macrophages.
4. Representative pictures macrophages differed from THP1.

**Figure S2**

1. Western blotting assays showing OI increased Nrf2 levels by impairing the interaction between KEAP1 and Nrf2 in BMDMs.
2. Western blotting assays showing OI increased Nrf2 levels in nucleus of BMEMs.
