# Supplementary material for: Overabundance of Veillonella parvula promotes intestinal inflammation by activating macrophages via LPS-TLR4 pathway
Source: Cell Death Discov. 2022 May 6;8:251. doi: 10.1038/s41420-022-01015-3 (PMC9076897; doi:10.1038/s41420-022-01015-3)
Supplement: Supplementary file 4 — table S1 [file 41420_2022_1015_MOESM4_ESM.docx]

**Table S1** Sequences of primers in mRNA expression analyses

|  | Sequences of forward primer | Sequences of reverse primer |
| --- | --- | --- |
| IL-1β (mouse) | TGGCAACTGTTCCTG | GGAAGCAGCCCTTCATCTTT |
| TNF-α (mouse) | GCCTCTTCTCATTCCTGCTT | TGGGAACTTCTCATCCCTTTG |
| IL-6 (mouse) | CTGCAAGAGACTTCCATCCAG | AGTGGTATAGACAGGTCTGTTGG |
| GAPDH (mouse) | TCCCACTCTTCCACCTTCGA | AGTTGGGATAGGGCCTCTCTT |
| IL-1β (human) | AGCTGATGGCCCTAAACACA | TGTCCATGGCCACAACAAC |
| IL-6 (human) | TCTGGATTCAATGAGGAGACTTG | CTCAAATCTGTTCTGGAGGTACT |
| TNF-α (human) | CCAGGGACCTCTCTC | TCAGCTTGAGGGTTT |
| GAPDH (human) | AGCCGAGCCACATCGCT | GCAACAATATCCACTTTAC |
